# Unexplained bleeding tendency and scleroderma-like skin changes: a diagnostic journey to vitamin C deficiency

**DOI:** 10.1016/j.rpth.2026.103424

**Published:** 2026-03-25

**Authors:** Florian Winkler, Peter Maximilian Heil, Johannes Thaler, Wolfgang R. Sperr, Cihan Ay, Johanna Strobl, Johanna Gebhart, Dino Mehic

**Affiliations:** 1 Department of Dermatology, Medical University of Vienna, Vienna, Austria; 2 Comprehensive Center for Inflammation and Immunity, Vienna, Austria; 3 Department of Medicine I, Division of Hematology and Hemostaseology, Medical University of Vienna, Vienna, Austria; 4 CeMM Research Center for Molecular Medicine of the Austrian Academy of Sciences, Vienna, Austria

**Keywords:** ascorbic acid deficiency, ecchymosis, hemorrhage, malnutrition, purpura

## Abstract

**Background:**

A subset of patients with a bleeding tendency undergoing hemostatic evaluation may be misdiagnosed as bleeding disorder of unknown cause (BDUC).

**Key Clinical Question:**

Should vitamin C deficiency be considered in patients with bleeding symptoms but normal hemostatic test results suggestive of BDUC?

**Clinical Approach:**

We report on a patient with bleeding symptoms despite normal hemostatic examination, in whom vitamin C deficiency was ultimately identified as the cause of the bleeding disorder. The patient, a 26-year-old man, presented with extensive ecchymoses, anemia, and sclerodermatous skin changes of the left leg. His nutritional anamnesis revealed markedly restricted eating habits. Vitamin C supplementation led to normalization of hemoglobin levels and marked improvement of the sclerodermatous skin changes.

**Conclusion:**

This case demonstrates that vitamin C deficiency can mimic hematologic and dermatologic disorders and highlights the importance of considering nutritional deficiencies as potential causes of bleeding, even in high-income countries.

## Essentials

• Patients bleeding despite normal tests may be labeled BDUC.
• A 26-year-old man presented with bruising, anemia, and skin thickening of the leg.
• Severe vitamin C deficiency was ultimately identified as the cause of the specific symptoms.
• Vitamin C testing might be considered in selected patients with BDUC when clinical suspicion is high.

## 1 Introduction

Patients presenting with a bleeding diathesis require comprehensive evaluation for acquired and hereditary disorders [1], yet a substantial proportion remain without a diagnosis despite thorough coagulation and platelet testing. These individuals are classified as having a bleeding disorder of unknown cause (BDUC), a diagnosis of exclusion that must also account for nonhemostatic causes [2].

Current International Society on Thrombosis and Haemostasis (ISTH) guidelines recommend considering acquired conditions [3], such as nutritional deficiencies [4], in the evaluation of unexplained bleeding, yet they are often overlooked in patients with BDUC. Leinøe et al. [5] recently showed that patients with BDUC have lower vitamin C levels than healthy controls. In a cross-sectional study of a German population, severe deficiency (<8.52 μmol/L) occurred in 3.3% [6], similar to rates in other high-income countries [7]. Long-standing, severe vitamin C deficiency impairs collagen hydroxylation by reducing prolyl and lysyl hydroxylase activity, leading to the clinical manifestations of scurvy in tissues with high collagen turnover, including skin, mucous membranes, and vascular walls. While scurvy typically presents with perifollicular bleeding, gingival hemorrhage, and poor wound healing, atypical manifestations can delay diagnosis [8,9]. We report a patient with unexplained bleeding and normal hemostatic testing, in whom vitamin C deficiency was ultimately identified as the likely underlying or contributing cause.

## 2 Case Presentation

A 26-year-old man presented to the emergency department of the University Hospital Vienna with numerous spontaneous hematomas, severe pain distributed over his entire body and a recent episode of self-limited epistaxis. He reported no trauma, anticoagulant use, liver disease, or known bleeding disorder and denied alcohol or drug use. Examination revealed extensive ecchymoses on the trunk and extremities, prominent bilateral popliteal hematomas, and hemorrhagic oral mucosal suffusions. Notably, he had marked swelling and induration of the left thigh with sclerotic skin changes and restricted knee mobility (Figure A–D). The patient presented with low body weight (body mass index of 18.8 kg/m^2^) and a history of anorexia nervosa in the past. A family history for bleeding was negative, neurological examination was normal, and a duplex ultrasound of the affected leg performed 10 days earlier excluded deep vein thrombosis.

**Figure:**
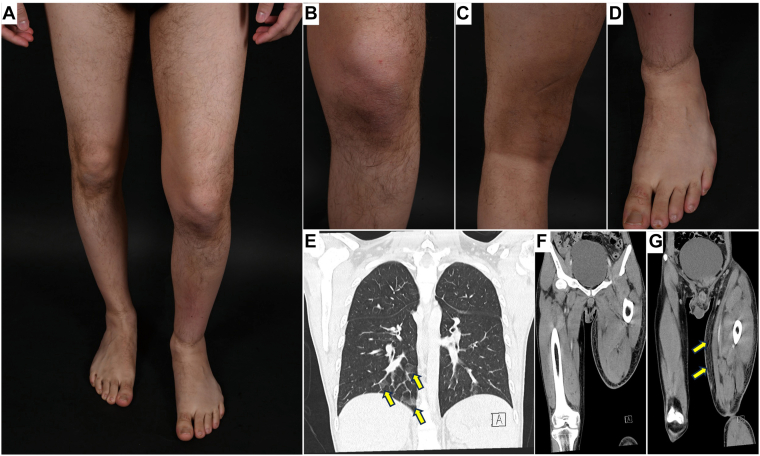
Sclerodermatous skin changes of the left leg and computed tomography (CT) scan. (A) Overview image and (B–D) close-ups demonstrating scleroderma-like skin changes of the left leg. (E–G) CT scan 2 days after hospitalization. (E) Lung CT shows ground-glass opacities in the lower lobes bilaterally. (F, G) CT of the lower extremities demonstrates diffuse edematous infiltration of the subcutaneous fat in the left thigh.

Initial blood work revealed a normocytic, normochromic anemia with a hemoglobin level of 7.8 g/dL (reference range, 13.5-18.0 g/dL), a normal platelet count of 229 × 10^9^/L (reference range, 150-350 × 10^9^/L), and leukocyte count of 5.15 × 10^9^/L (reference range, 4-10 × 10^9^/L). Apart from a slightly elevated C-reactive protein level of 3.12 mg/dL (<0.5 mg/dL), serum chemistry parameters showed no abnormalities. Vitamin B12, folic acid, and iron parameters were within in the normal range. A broad coagulation workup was performed, which showed prolongation of both prothrombin time (55%; reference range, 70%-125%) and activated partial thromboplastin time (44 seconds; reference range, 27-41 seconds). Fibrinogen was markedly elevated at 565 mg/dL (reference range, 200-400 mg/dL). A lupus anticoagulant assay was negative. Repeated testing confirmed these abnormalities. Individual factor assays revealed mildly reduced factor (F)V (60%; reference range, 75%-130%) and FVII (54%; reference range, 75%-160%) activities insufficient, however, to account for the bleeding phenotype. In contrast, FVIII (257%; reference range, 60%-140%) and FIX (163%; reference range, 60%-140%) activities were distinctly elevated. There was no evidence of FXIII deficiency. von Willebrand factor antigen (134%) and activity (169%) were within normal limits. Protein C and protein S levels were normal, and plasminogen activator inhibitor-1 activity was not reduced either.

To exclude active bleeding and other underlying diseases, a contrast-enhanced computer tomography scan of the thorax, abdomen, and lower extremities was performed, which showed diffuse edematous infiltration of the subcutaneous fatty tissue in the left thigh and ground-glass opacities in the lower lobe on both sides, findings that were most consistent with hemorrhages in the given clinical context (Figure E–G). No active bleeding source was found. Thus, also in view of the unremarkable hematinics, the cause of anemia remained unclear. However, given the extent of soft tissue hemorrhage and repeated spontaneous hematomas, blood loss in the context of severe vascular fragility due to vitamin C deficiency was considered the most plausible explanation. Due to the sclerotic skin changes, the patient was referred to the department of dermatology, where a skin biopsy revealed unspecific perivascular and interstitial lymphocytic inflammation.

In view of the patient’s history of anorexia nervosa and low body mass index, a dietary consultation was obtained, which revealed orthorexic eating behavior with a highly restrictive and unbalanced diet consisting almost exclusively of white bread. This prompted measurement of vitamin C, which was found to be undetectable (<2.27 μmol/L; reference range, 26.1-84.6 μmol/L). With a normal hemostatic workup, the bleeding and sclerotic skin changes were attributed to severe vitamin C deficiency consistent with the clinical manifestations of scurvy. Screening for inherited syndromal disorders or platelet aggregometry was not performed due to limited resources and the strong clinical and laboratory evidence supporting vitamin C deficiency as the primary cause of the patient’s symptoms.

In view of the severe anemia, the patient received a total of 6 erythrocyte concentrates, which led to an increase in hemoglobin to 10.0 g/dL. Following the diagnosis of scurvy, appropriate supplementation therapy was implemented, leading to a normalization of vitamin C and hemoglobin levels to 93.5 μmol/L and 13.9 g/dL, respectively, at the last control. This was accompanied by marked improvement of the sclerodermatous skin changes.

## 3 Discussion

We report a patient in whom vitamin C deficiency was the primary cause of the bleeding tendency. Although uncommon in high-income countries, vitamin C deficiency continues to occur in certain risk groups, particularly in individuals with restricted diets, alcohol use disorder or malabsorption syndromes [[10], [11], [12], [13]]. In our patient, severely reduced vitamin C level was caused by a diet consisting almost exclusively of white bread, underlining the importance of a detailed nutritional anamnesis in patients with unexplained bleeding [1]. This is consistent with a recent Danish study, demonstrating that many patients with BDUC exhibit reduced or suboptimal vitamin C levels compared with healthy controls [5].

Vitamin C is an important micronutrient with a wide range of physiological roles. By acting as a cofactor for the hydroxylation of proline residues in procollagen, vitamin C is essential for the synthesis and stabilization of the collagen triple helix [14]. Insufficient vitamin C status therefore impairs collagen synthesis, contributing to vascular fragility and, consequently, the formation of hematomas and other bleeding manifestations [15]. Importantly, vitamin C deficiency–associated bleeding may occur despite inconclusive coagulation parameters, as observed in our patient [16]. Furthermore, reduced vitamin C levels have been reported in individuals with bleeding tendencies carrying germline variants of uncertain significance in collagen-related genes [17]. Interestingly, the above-mentioned Danish study of 60 patients with BDUC reported an association between low vitamin C levels and joint hypermobility [5]. Nevertheless, our patient did not display features suggestive of connective tissue disease, such as hypermobility or skin fragility, making a hereditary collagen disorder (eg, Ehlers–Danlos syndrome) unlikely. Therefore, further genetic testing was not pursued.

Beyond its hematologic manifestations, vitamin C deficiency can also affect the skin. The characteristic dermatologic findings primarily reflect the underlying bleeding tendency, ranging from petechiae and perifollicular hemorrhages up to extensive ecchymoses, often with a predilection for the lower extremities. Additional manifestations include follicular hyperkeratosis and the presence of “corkscrew” and “swan-neck” hairs [18,19]. These hair abnormalities occur because ascorbic acid plays a crucial role in the disulfide bonding necessary for normal hair formation. Consequently, vitamin C deficiency can lead to fragile, fractured hairs that coil into corkscrew or swan-neck configurations [20].

As illustrated by our case, vitamin C deficiency can also present with sclerotic skin changes, an uncommon manifestation first described by Grusin and Kincaid-Smith in 1954 [21]. Subsequent reports have described sclerodermatous changes in vitamin C–deficient patients, typically affecting the lower extremities with swelling, induration, and reduced mobility. Histopathology, including in our 26-year-old patient, is usually nonspecific, showing perivascular and interstitial lymphocytic inflammation, erythrocyte extravasation, and dermal hemosiderin deposits [22,23]. It has been proposed that hemosiderin deposition drives fibroblast proliferation and dermal sclerosis, supported by experimental data from Golberg et al. [24], where subcutaneous iron injections in mice induced siderophage infiltration and fibrotic reactions [24].

The present case underscores the importance of measuring vitamin C in unexplained bleeding with normal hemostatic tests. Vitamin C deficiency represents a readily measurable condition and an easily excluded diagnosis in such cases. However, it remains a relatively rare cause of BDUC among numerous other potential etiologies, including disorders of platelet function, fibrinolysis, the tissue factor pathway inhibitor, or activated protein C, as well as diseases affecting vascular integrity and gap junctions. Routine screening for vitamin C deficiency in all patients with BDUC therefore remains questionable. The assessment of vitamin C status may be most appropriate in selected patients with risk factors such as restricted diets, malnutrition, malabsorption, alcohol use disorder, or suggestive dermatologic features. Further studies are needed to define which patient subsets benefit most from nutritional testing and to clarify the contributory role of vitamin C deficiency within the spectrum of BDUC.
